# Absorption–Reflection–Transmission Power Coefficient Guiding Gradient Distribution of Magnetic MXene in Layered Composites for Electromagnetic Wave Absorption

**DOI:** 10.1007/s40820-025-01675-7

**Published:** 2025-02-17

**Authors:** Yang Zhou, Wen Zhang, Dong Pan, Zhaoyang Li, Bing Zhou, Ming Huang, Liwei Mi, Chuntai Liu, Yuezhan Feng, Changyu Shen

**Affiliations:** 1 State Key Laboratory of Structural Analysis, Optimization and CAE Software for Industrial Equipment, National Engineering Research Center for Advanced Polymer Processing Technology, Zhengzhou University, Zhengzhou, 450002 People’s Republic of China; 2 Yaoshan Laboratory, Pingdingshan University, Pingdingshan, 467000 People’s Republic of China

**Keywords:** Magnetic MXene, Layered and gradient structure, Power coefficient, Electromagnetic wave absorption

## Abstract

The morphological distribution of absorbent in composites is equally important with absorbents for the overall electromagnetic properties, but it is often ignored. Herein, a comprehensive consideration including electromagnetic component regulation, layered arrangement structure, and gradient concentration distribution was used to optimize impedance matching and enhance electromagnetic loss. On the microscale, the incorporation of magnetic Ni nanoparticles into MXene nanosheets (Ni@MXene) endows suitable intrinsic permittivity and permeability. On the macroscale, the layered arrangement of Ni@MXene increases the effective interaction area with electromagnetic waves, inducing multiple reflection/scattering effects. On this basis, according to the analysis of absorption, reflection, and transmission (A–R–T) power coefficients of layered composites, the gradient concentration distribution was constructed to realize the impedance matching at low-concentration surface layer, electromagnetic loss at middle concentration interlayer and microwave reflection at high-concentration bottom layer. Consequently, the layered gradient composite (LG5-10–15) achieves complete absorption coverage of X-band at thickness of 2.00–2.20 mm with *RL*_min_ of −68.67 dB at 9.85 GHz in 2.05 mm, which is 199.0%, 12.6%, and 50.6% higher than non-layered, layered and layered descending gradient composites, respectively. Therefore, this work confirms the importance of layered gradient structure in improving absorption performance and broadens the design of high-performance microwave absorption materials.

**Supplementary Information:**

The online version contains supplementary material available at 10.1007/s40820-025-01675-7.

## Highlights

- The layered arrangement and gradient distribution of magnetic MXene are firstly combined to improve the electromagnetic wave (EMW) *RL*_min_ and broaden effective absorption bandwidth.
- Absorption, reflection, and transmission (A–R–T) power coefficient analysis is firstly used to guide the gradient distribution, so as to realize EMW incidence at low-concentration surface, loss at middle concentration interlayer and reflection at high-concentration bottom.
- The layered gradient composite (LG5-10-15) achieves complete absorption coverage of X-band at thickness of 2.00-2.20 mm with *RL*_min_ of -68.67 dB.

**Supplementary Information:**

The online version contains supplementary material available at 10.1007/s40820-025-01675-7.

## Graphical abstract

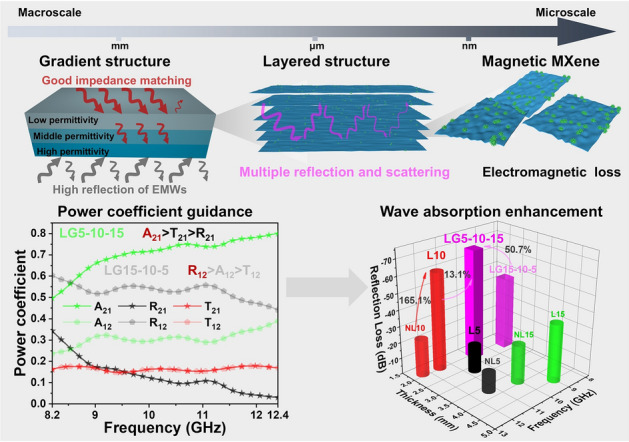

## Introduction

The widespread use of communication technologies has created a growing demand for electromagnetic wave (EMW) absorbing materials that eliminate undesired electromagnetic radiation and interference, ensuring the appropriate operation of advanced precision equipment [1–3]. In particular, the cutting-edge skins of stealth aircraft, military missiles, and precision instruments also require EMW absorbing materials with integrated multifunctional properties that significantly improve their survivability and combat efficiency on the battlefield [4–6]. It is well known that the microwave absorption performance of the material is determined by the electromagnetic parameters including intrinsic permittivity and permeability, which are mainly dependent on the components and microstructure of absorbents [7, 8]. However, in practical applications such as coatings or hybrid shells, the morphological distribution of the absorbent in carriers also has a significant impact on the overall electromagnetic properties of the coatings or composites and then has an important impact on the EMW absorbing properties [9–11], but it is often ignored.

At present, the various absorbents based on carbon-based materials (graphene, carbon fiber, and carbon nanotubes) [12–14], magnetic-based materials (ferrite, alloys, and metallic oxides) [15–18], and conductive polymers [19, 20] have been developed. Recently, the transition metal carbide/carbon nitride material (MXene) has emerged as ideal candidate for electromagnetic protective materials due to its unique two-dimensional (2D) laminated structure, rich surface polar groups, and adjustable dielectric properties [21–23]. However, the metal-level conductivity derived from the metallic Ti layer on the surface of MXene causes a large amount of EMW reflection; thus, MXene-based materials are typically employed in electromagnetic shielding rather than electromagnetic absorption [24–26]. Fortunately, the incorporation of magnetic components into MXene nanosheets can effectively regulate their dielectric properties to the suitable range for impedance matching and meanwhile endow the magnetic loss ability for broadening the absorption bandwidth, thereby achieving excellent EMW absorbing capabilities. The previous studies have constructed various magnetic heterostructures of MXene/Ni [27], Ni/NC@Ti_3_C_2_T_x_ [28], MXene/NiCo_2_S_4_ [29], and MXene@Ni [30], achieving electromagnetic synergistic loss capabilities for the development of MXene-based absorbing materials. The current researches of MXene-based absorbing materials mainly focus on the regulation of electromagnetic components and the design of microstructure. In practical applications, powder absorbents, including magnetic MXene hybrids, are usually combined with adhesives and coated on the surface of protected objects [31, 32]. However, this category coatings with randomly dispersed absorbents mainly face the two problems of agglomeration and high content [33, 34]. The former results in local conduction that affects the electromagnetic loss capacity, and the latter significantly increases the associated cost.

By contrast, the morphological distribution of absorbents in carriers (coatings or composites) is particularly important in improving the microwave absorbing ability of integral absorbing materials (containing absorbents and matrix). By orderly distributing the absorbents in matrix, conductive networks with hierarchical interfaces can be regularly formed at the low filler loading, which extend the EMW transmission path by multiple reflection/scattering, thus enhancing the EMW loss ability [35, 36]. For example, Chen et al. [37] carried out a comparative study on the influence of random versus orientation distribution of MXene in epoxy resin on EMW absorption properties and found that orientation distribution could significantly improve permittivity, dielectric loss, reflection loss (*RL*), and effective absorption bandwidth (EAB). Our previous work [38] also revealed that regulating the integral alignment of Ni@MXene in layered composites could significantly improve the EMW absorption performance with the *RL*_min_ of −69.8 dB and EAB of 4.77 GHz. According to the mechanism analysis, the layered orientation distribution of Ni@MXene increased the contact area between the absorbents and EMWs, induced multiple reflection/scattering effects, and formed local conduction loss, thus increasing the EMW loss ability of layered composites. However, the layered composites often show a narrow EAB due to the facilitated formation of conductive networks by orderly aligning absorbents. In order to broaden the EAB, it is first required that the surface of absorbing composite exhibits optimal impedance matching conditions to allow more EMWs to enter the interior. At the same time, the interior of absorbing composite must pose high loss capacities to ensure the strong absorption of EMWs [39–41]. As a result, the gradient structure designed with low-concentration surface layer as impedance matching layer, medium-concentration core layer as absorbing layer, and high-concentration bottom layer as reflecting layer emerges. Therefore, as stated in the above, the comprehensive design of layered arrangement and gradient concentration distribution of 2D magnetic MXene in composite is promising to achieve wide and strong EMW absorption, but not yet reported.

In this work, we have integrated electromagnetic component regulation, layered structure design, and gradient concentration distribution to optimize impedance matching, extend EMW transmission paths, and enhance electromagnetic synergistic loss to reinforce *RL* and broaden EAB. Firstly, magnetic Ni nanoparticles were introduced onto the surface of MXene to regulate its electromagnetic properties. Then, layered oriented arrangement of Ni@MXene absorbent was achieved by hot pressing with high compression ratio. Finally, the layered composites with different Ni@MXene content were interface-melt-welded to each other, and the gradient concentration was determined according to the qualitative analysis of A–R–T coefficients of each layer. Moreover, poly(vinylidene fluoride-co-hexafluoropropylene) (PVDF-HFP) with polar C–F bond was selected as the ideal matrix for absorbing materials compared to other polymers [42–44]. As anticipated, the layered ascending gradient composite (LG5-10–15) not only exhibits superior reflection loss capability with *RL*_min_ of -68.67 dB at 9.85 GHz in 2.05 mm, but also achieves full coverage of the X-band frequency range within the thickness of 2.00–2.20 mm. In contrast, under the same Ni@MXene content, the *RL*_min_ of layered ascending gradient composites is increased by 199.0%, 12.6%, and 50.6% compared with those of non-layered (NL10), layered (L10), and layered descending gradient (LG15-10–5) composites. Overall, this paper strongly confirms the effectiveness of layered and gradient structure in enhancing absorption intensity and broadening absorption bandwidth.

## Experimental Section

### Materials

Ti_3_AlC_2_ powders (MAX, 200 mesh) were purchased from Jilin 11 Technology Co., Ltd. (China). Nickel chloride hexahydrate (NiCl_2_·6H_2_O), hydrazine hydrate (N_2_H_4_·H_2_O), sodium hydroxide (NaOH), ethylene glycol, alcohol, hydrochloric acid (HCl), and polyethylene glycol (PEG, 10,000 g mol^−1^) were purchased from Sinopharm Chemical Reagent Co. Ltd. (China). N, N-dimethylformamide (DMF) and lithium fluoride (LiF) were purchased from Aladdin Reagent Co., Ltd (China). PVDF-HFP were supplied by Beijing InnoChem Science & Technology Co., Ltd. (China). Deionized water (DI) was laboratory-made, and all chemical reagents were used without further processing.

### Preparation of Ti_3_C_2_T_x_ MXene

LiF (5 g) was thrown into the Teflon beaker containing 100 mL HCl (9 mol mL^−1^) at 35 °C and by stirring smoothly for 30 min. Immediately, MAX (5 g) was slowly added to the solution; afterward, the aluminum atom etching reaction of MAX was maintained at 35 °C for 24 h under stirring, after which the obtained Ti_3_C_2_T_x_ solution was washed with DI by centrifuged (3500 rpm) several times, until its pH reached 5–6. The sediment was ultrasonically exfoliated and shaken with alcohol for 1 h, and centrifuged at 10,000 rpm for 10 min. Finally, the colloidal suspension containing 2D sheetlike MXene was collected multiple times by centrifugation at 3500 rpm for 3 min and manual agitation.

### Synthesis of Magnetic Ni@MXene Absorbent

The successful preparation of Ni@MXene absorbent was achieved by modifying MXene with magnetic Ni particle based on previous experiments [38]. Firstly, NiCl_2_·6H_2_O (2.015 g) was completely dissolved in DI to form an aqueous solution of Ni^2+^. Subsequently, a water solution containing 0.5 g of MXene was added to 225 mL of DI and 225 mL of EG, and then, NaOH (4.0 g) was added to create an alkaline environment. After that, Ni^2+^ solution was added to the above alkaline MXene solution and stirred for 15 min, relying on charge adsorption to allow positively charged Ni^2+^ to adsorb on the surface of negatively charged MXene. Finally, the reductant N_2_H_4_·H_2_O (10.5 mL, 80 wt%) was slowly added into the above mixture and fully stirred for 15 min, and then transferred to a water bath at 60 °C for 2 h. The resultant Ni@MXene was collected by static settlement and replaced into an organic DMF solution.

### Fabrication of Layered and Gradient Ni@MXene/PH Composites

**Fig. 1:**
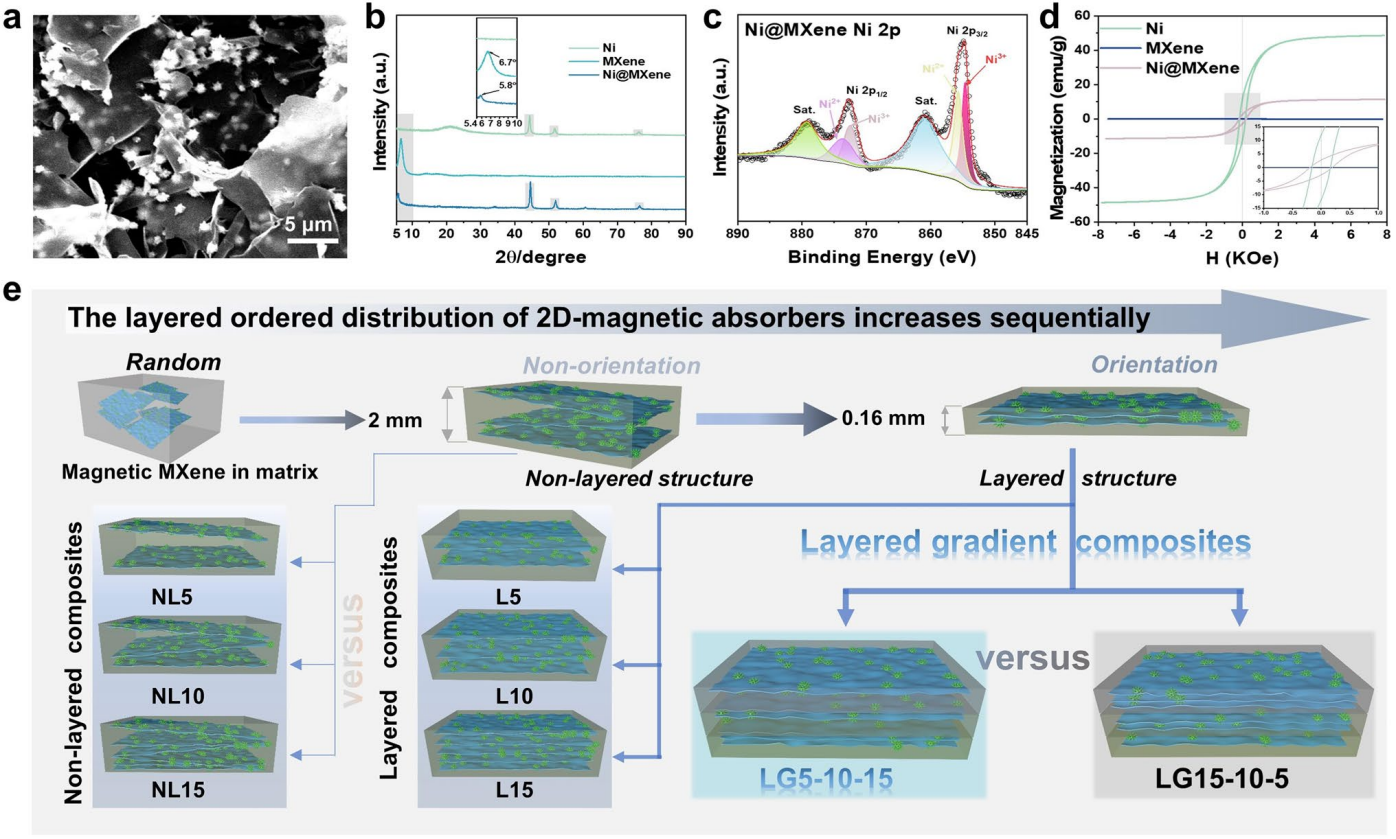
**a** SEM image of Ni@MXene absorbent, **b** XRD patterns of Ni, MXene, Ni@MXene, **c** XPS spectra of the absorbent Ni@MXene at Ni 2*p* region, **d** magnetic hysteresis loops of Ni, MXene, and Ni@MXene at room temperature. **e** Schematic illustration of fabricating layered and gradient Ni@MXene/PH composites

The layered gradient Ni@MXene/PH composites were fabricated by hot pressing-induced alignment of magnetic MXene nanosheets and gradient stacking of different absorbent contents (Fig. 1e). Firstly, an appropriate amount of PVDF-HFP was dissolved into DMF at 60 °C for 2 h. Secondly, the Ni@MXene absorbent solution and PEG were evenly dispersed into the PVDF-HFP solution by mechanical agitation about 2 h. (The mass fraction of Ni@MXene absorbent is 5, 10, and 15 wt%, respectively.) After that, the Ni@MXene/PH (the filler is Ni@MXene, and the matrix is PVDF-HFP) composites were obtained through water-induced phase separation technology and dried at 60 °C in air to remove moisture. The layered structure of magnetic MXene nanosheets in matrix was achieved by step-by-step hot pressing with mold thickness of 0.16 mm, respectively, at 200 °C for 10 min under 2.60 MPa pressure. The non-layered Ni@MXene/PH composites (2.0 mm mold thickness) and layered Ni@MXene/PH composites (2 mm mold thickness) were named as NL5, NL10, NL15 and L5, L10, L15, respectively, according to their filler loading. The layered gradient Ni@MXene/PH were prepared through stacking L5, L10, and L15 (4 layers each) of hot pressing process in sequence, and named LG5-10–15 and LG15-10–5 composites according to the incidence direction of EMWs.

### Characterizations

The morphology, microstructure, and distribution of elements of composites were observed by the scanning electron microscope (SEM JSM-7001F, Zeiss Sigma 300) equipped with an energy-dispersive spectrometer. Orientation degree of magnetic MXene nanosheets was characterized by 2D wide-angle X-ray diffraction (A Bruker D8 Discover diffractometer, 2D-WAXD). The crystal and chemical structure were obtained via X-ray diffraction (XRD, Rigaku Ultima IV, Cu Kα radiation, *λ* = 0.154 nm), Fourier transform infrared spectrometer (FTIR, Nicolet 6700), and X-ray photoelectron spectroscopy (XPS, Al Kα, Thermo ESCALAB 250XI). The magnetic property was tested by a vibrating sample magnetometer (VSM, LakeShore 7404). The tensile test was carried out on a universal tensile testing machine (Shimadzu, Japan).

To measure the electromagnetic microwave absorption performance, the composites, each 2 mm in thickness, were carved into a rectangle with a length of 22.9 mm and a width of 10.2 mm using the carving machine (G4030 three-axis computer engraving machine). The scattering parameters and electromagnetic parameters were measured using the waveguide on a vector network analyzer (Keysight Technology N5222B) in the frequency range of 8.2–12.4 GHz. The incident wave of the vector network analyzer penetrated vertically into the plane of the rectangle during the testing process. The A, R, T, and the electromagnetic interference shielding effectiveness (EMI SE) were obtained based on the measurements of the scattering parameters. (Details are shown in Section S2.) The electromagnetic microwave absorption performances were calculated from the measured electromagnetic parameters based on the transmission line theory. (Details are shown in Sections S3 and S4.)

## Results and Discussion

### Preparation and Characterizations of Layered and Gradient Composites

The research purpose of this work is to investigate the effect of the layered ordered structure and gradient distribution of 2D magnetic MXene absorbent on their microwave absorption performance. First of all, in order to solve the problem of impedance mismatch and introduce the magnetic loss capacity for the composite, magnetic Ni@MXene was successfully prepared with Ni nanoparticles riveting MXene (Figs. 1a and S1). The XRD characteristic diffraction peaks of Ni in the (111), (200), and (220) crystal planes and the obvious (002) diffraction peak shifts from 2θ = 6.7 to 2θ = 5.8° of MXene (Fig. 1b) and the XPS peaks of Ni 2*p* including Ni 2*p*_1/2_ (core level 872 eV, satellite characteristics 878.9 eV) and Ni 2*p*_3/2_ (core level 855 eV, satellite characteristics 860.8 eV) in Figs. 1c and S2a effectively indicate that magnetic metal Ni has been successfully riveted onto MXene [27, 45]. The shifts of Ti–O, C = O, and Ti–C bonds fitting peaks in the Ti 2*p*, O 1*s*, and C 1*s* XPS spectra of Ni@MXene compared to MXene (Fig. S2b-d), as well as the shift of -OH absorption peak [46] in its FTIR (Fig. S3), suggest the electrostatic interaction between Ni and MXene. Besides, the abundant fitting peaks in XPS spectra and absorption peaks in FTIR spectra of Ni@MXene reveal that the Ni@MXene surface still retains numerous functional groups [47], which not only help to improve the interface compatibility with matrix, but also produce a lot of dipole polarization in the electromagnetic field. The magnetic Ni endows the absorbent Ni@MXene with strong ferromagnetic properties (Fig. 1d), with saturation magnetization (Ms), residual magnetization (Mr), and coercive force (Hc) reaching 11.58 emu g^−1^, 2.65 emu g^−1^, and 190 Oe, respectively. The strong magnetism makes Ni@MXene easily adsorbed by a magnet compared to MXene (Fig. S4). In this work, the addition of magnetic particles is expected to regulate impedance matching, and provide the magnetic loss capability for the absorbing material.

The process for manufacturing layered gradient structured Ni@MXene/PH composites is illustrated in Fig. 1e. The solution mixing of Ni@MXene was evenly dispersed in PVDF-HFP matrix, where a small amount of PEG was used to improve the compatibility. After phase separation, the non-layered Ni@MXene/PH composites (NL5, NL10, and NL15) were prepared by hot pressing using the 2.0 mm thick mold, in which the thicker frame is not sufficient to induce shear flow of inducing in-plane orientation. In order to improve the orientation of the 2D Ni@MXene absorbent to form the layered structure in composites, the non-layered Ni@MXene/PH composites with an initial thickness of 2.0 mm were further hot-pressed to achieve the final thickness of 0.16 mm. During this process, the high compression ratio can induce in-plane shear flow in the melt, which induces 2D Ni@MXene to highly align along in-plane orientation [48]. The layered Ni@MXene/PH composites (L5, L10, and L15) were prepared by hot pressing the above-mentioned 0.16 mm oriented material. To further investigate the impact of Ni@MXene gradient concentration on the microwave absorption performance of composites, the layered structure composites (L5, L10, and L15) were interface-melt-welded to each other using hot pressing without damaging the layered structure. Based on the different directions of EMW incidence, it can be divided into ascending and descending concentration layered gradient composites with the thickness of 2.0 mm, which are named LG5-10–15 and LG15-10–5 in sequence.

**Fig. 2:**
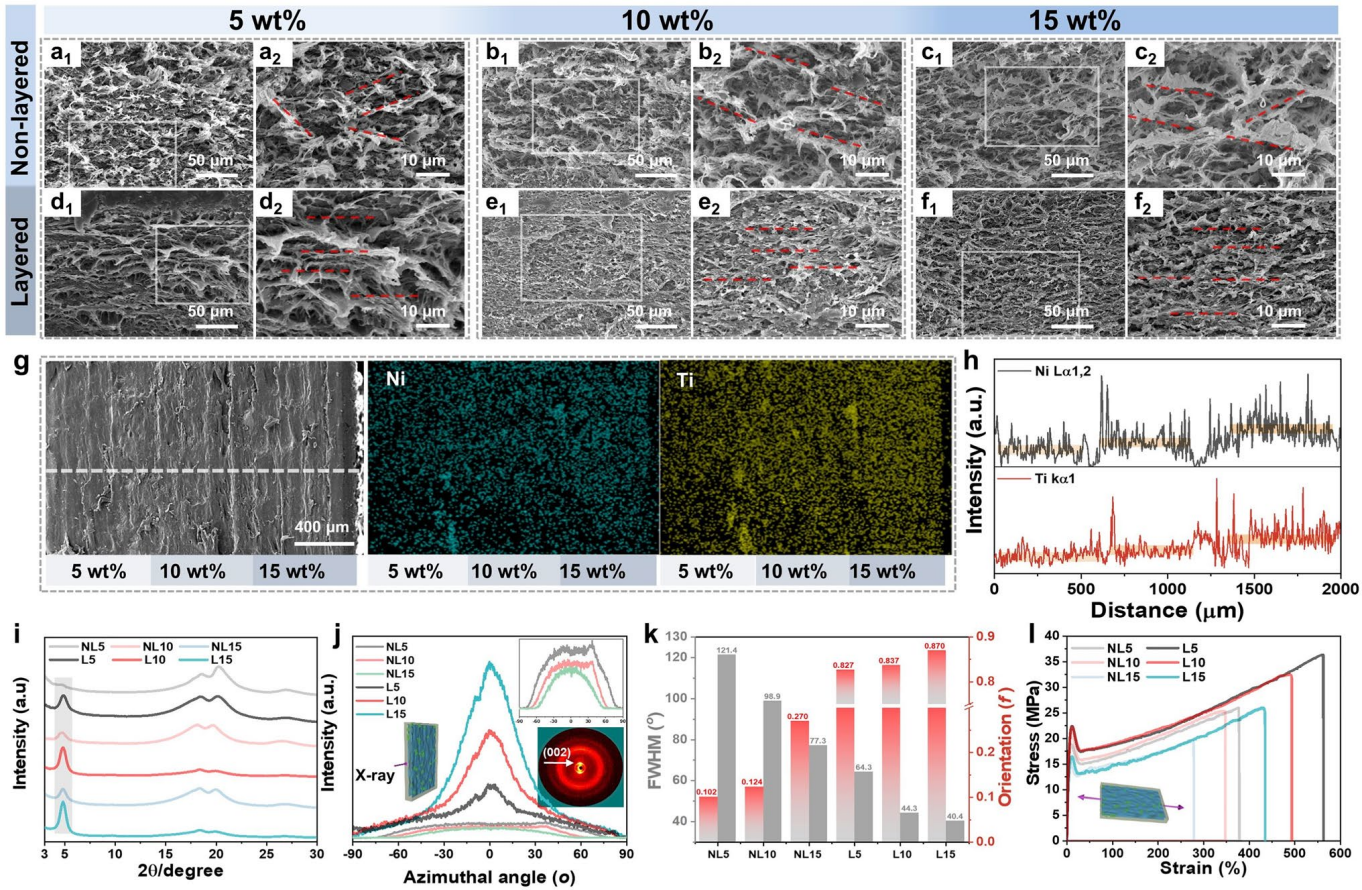
SEM images of the cross-sectional morphologies for **a** NL5, **b** NL10, **c** NL15, **d** L5, **e** L10, **f** L15 composites. **g** Cross-sectional SEM image, corresponding element mapping images, and **h** energy-dispersive X-ray spectroscopy line scanning of layered gradient structured Ni@MXene/PH composite. **i** XRD, **j** 2D-WAXD and their corresponding **k** FWHM and orientation factor f, and **l** stress–strain curves of NL5, NL10, NL15, L5, L10, L15 composites; inset in **j** is 2D-WAXD image of L15

The layered and gradient structures of the Ni@MXene/PH composites are verified in Fig. 2. The cross-sectional SEM morphologies shown in Fig. 2a-c reveal a disorder distribution of Ni@MXene nanosheets in the NL samples, which can be attributed to the low compression ratio during hot pressing. The distribution can be easily changed from the original disorder to the layered ordered distribution (Fig. 2d-f) by further improving the compression ratio. During hot pressing with frame thickness of only 0.16 mm, the pressure of the hot press can be effectively converted into shear force during the melt flowing state, thus inducing the nanosheets to be oriented and orderly distributed along in-plane direction. With the increase in the filler content, the layered structure of the composite becomes more obvious due to the crowding stacking effect between the nanosheets. The cross-sectional SEM morphology of LG5-10–15 (Fig. 2g) shows obvious but dense weld marks without defect, suggesting the well and intact construction of layered and gradient structure by simple interfacial molten welding. The gradient distribution is confirmed by energy-dispersive X-ray surface line scanning analysis. As shown in Fig. 2g, h, the content of both nickel and titanium elements exhibits the increasing gradient trend from left to right according to the cross-sectional image, which indicates successful fabrication of the gradient structure of composite.

Furthermore, due to the anisotropic crystal structure of MXene, the orientation distribution of Ni@MXene in the composites can be characterized by XRD. As shown in Fig. 2i, the stronger intensity of the (002) diffraction peak of MXene at 2θ = 5° in layered series composites when compared to non-layered series composites clearly indicates that the nanosheets have achieved layered arrangement by high compression ratio hot pressing treatment. To further quantify the orientation degree of the MXene in layered composites, the 2D-WAXD analysis was conducted. As shown in the azimuthal plots in Fig. 2j, the layered series exhibit more obvious peaks [49] with narrower full width at half maximum (FWHM) when compared to the non-layered series. The more obvious fan-shaped bright spot (7–9 Å^−1^) is shown in the inset in Fig. 2j compared to Fig. S5. The orientation factor (f) can be calculated using the Herman's orientation formulas S1-S2 in Supporting Information, and are presented in Fig. 2k. It can be found that the orientation degrees of Ni@MXene in layered series composites are greatly larger than those in non-layered series composites; for example, the f value of L15 is 0.870 while this value of NL15 is only 0.102. In addition, the orientation factors of both layered series and non-layered series composites show a continuously increasing trend with the increase in the Ni@MXene content, which is opposite to the trend of FWHM.

The in-plane orientation arrangement of nanosheets is helpful to enhance the mechanical properties of the composites. As shown in Fig. 2l, all samples exhibit typical stress–strain curves of ductile fracture when stretched along the in-plane orientation direction. In comparison, the layered series composites show better tensile strength, fracture strain, and toughness than the non-layered series (Fig. S6). The enhanced mechanical properties are primarily attributed to the highly ordered layered distribution of the filler, which effectively transmits internal stress to the matrix, preventing the occurrence of micro-cracks [50, 51]. However, different from the trend of f with increasing filler content, the mechanical properties show the opposite trend with optimal tensile strength, fracture strain, and toughness of 36.28 MPa, 562.6% and 151.7 MJ m^−3^ for L5, which may be due to the more defects in composites with higher filler loading. Besides, the magnetic MXene also endows the layered composites with ferromagnetic properties (Fig. S7) that were gradually enhanced with increasing filler loading. The layered gradient (LG) composite with moderate saturation magnetization and lower coercive force is between L10 and L15, indicating that the LG composite has certain magnetic attenuation properties toward EMWs [52–54].

### A–R–T Coefficients and Electromagnetic Parameters of Layered and Gradient Composites

**Fig. 3:**
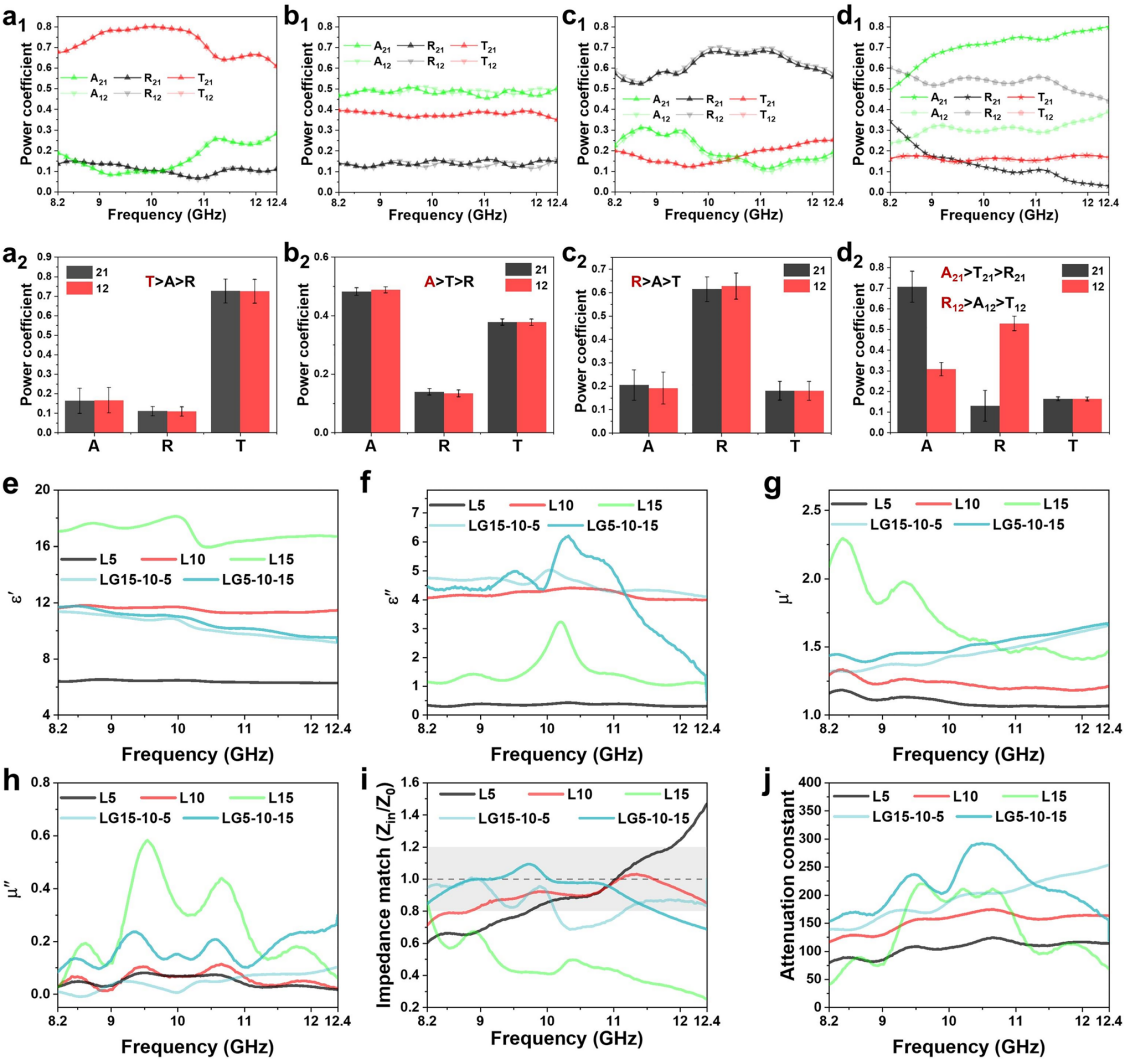
A–R–T coefficients and their corresponding average statistical values of **a** L5, **b** L10, **c** L15, **d** LG15-10–5 (port II), and LG5-10–15 (port I) composites. **e** Real part permittivity, **f** imaginary part permittivity, **g** real part permeability, **h** imaginary part permeability, **i** impedance matching, and **j** attenuation constant of L5, L10, L15, LG15-10–5, and LG5-10–15 composites

In order to confirm the directional EMW absorption performance of layered gradient Ni@MXene/PH composites, the scattering parameters were tested by using the vector network analyzer by the waveguide method; the absorption (A), reflection (R), and transmission (T) coefficients, and SE_A_, SE_R_, SE_T_ of port I and port II were calculated using formulas S3-S10. The EMI SE of the layered and non-layered Ni@MXene/PH composites shows a gradually increasing trend with increasing filler content (Figs. S8 and S9), but their values are less than 10 dB. Due to the uniform distribution of Ni@MXene absorbent in these composites, the A–R–T coefficients of port I and port II almost completely overlap (Figs. 3a-c and S9g-i). With increasing the filler loading, the relationship between A–R–T changes from T > A > R (L5, NL5) to A > T > R (L10, NL10), and R > A > T (L15, NL15). This shows that the impedance matching of the composites changes from weak to suitable to strong, and the internal absorption loss ability is gradually strengthened. Therefore, the composite with the layered gradient structure is arranged in the sequence of LG5-L10-L15 with L5 as incident layer, L10 as loss layer, and L15 as reflection layer. As expected, due to the gradient increased Ni@MXene content in layered gradient composites, the A–R–T coefficients of port I and port II exhibit significant differences with the relationships of A_21_ > T_21_ > R_21_ and R_12_ > A_12_ > T_12_, respectively (Fig. 3d), which effectively illustrates the gradient structure properties of the composites. At port I, most EMWs pass through transmission layer (L5) with little reflection, and the majority of EMWs interact with the absorbents in loss layer, while a minimal fraction of EMWs is reflected back to loss layer by reflection layer (L15) and subsequently consumed. Consequently, LG5-10–15 can achieve the maximum possible absorption of EMWs. However, in the case of LG15-10–5 at port II, the majority of EMWs are reflected by the reflection layer, failing to effectively utilize the absorption capacity of the absorbent in the intermediate loss layer.

To investigate the electromagnetic absorption properties of layered and gradient composites, the relative complex permittivity (***ε***_*r*_ = *ε′-jε″*) and the relative complex permeability (***μ***_*r*_ = *μ′-jμ″*) were obtained from scattering parameters (S_11_, S_21_) measured in the range from 8.2 to 12.4 GHz using the waveguide method. (The detailed inference formula can be found in reference [55].) As shown in Figs. 3e, f and S10a, S11a-c, the *ε′* exhibits an increasing trend with the increase in the absorbent content in layered or non-layered composites due to the increasing conductive network, while *ε″* and Tan*δ*_*ε*_ reveal a trend of increasing first and then decreasing with L10 and NL10 showing the best loss ability. In contrast, the layered samples show higher *ε′* and *ε″* than the non-layered samples due to the easy formation of conduction network for orientation Ni@MXene nanosheets. For the *μ′*, *μ″*, and Tan*δ*_*μ*_, they show a continuous increasing trend with the increase in the absorbent content (Figs. 3g-h and S10b, S11d-f), which mainly depends on the ferromagnetic strength of the composites. Interestingly, it can be seen that LG15-10–5 and LG5-10–15 reveal the similar *ε′* values between 8 and 12 with decreasing trend as the increase in the frequency (Fig. 3e), which is a normal range of microwave absorbing materials and can be explained by the polarization hysteresis behaviors at high frequency [56, 57]. The *ε′*, *ε″*, and Tan*δ*_*ε*_ of LG15-10–5 and LG5-10–15 appear obvious peaks within 9–11 GHz range, which may be due to the existence of complicated polarization relaxation and polarization phenomena when EMWs enter composites. According to the relative content of the absorbent, the complex magnetic permeabilities of LG5-10–15 and LG15-10–5 are lower than those of composites L15 and close to those of composites L10 (Fig. 3g, h). Besides, there are many obvious resonance peaks in *μ″–f* and Tan*δμ–f* curves of LG5-10–15 composite due to the magnetic resonance effect of magnetic Ni@MXene under electromagnetic field [58]. In comparison, LG5-10–15 manifests superior *ε′*, *ε″*, Tan*δ*_*ε*_, and *μ′*, *μ″*, Tan*δμ* values than those of LG15-10–5, which means that the layered ascending gradient structure is more conducive to the effective absorption of EMWs compared to the layered descending gradient structure.

To obtain excellent microwave absorption performance, impedance matching (**Z**_in_/**Z**_0_) that can be calculated by formula S11 is usually the first metric to be considered, because it determines how much EMW enter materials. In the case of layered composites (Fig. 3i), L10 exhibits the best impedance matching (around 1 at full frequency) compared to L5 and L15 due to its suitable permittivity. For layered gradient composites, **Z**_in_/**Z**_0_ values of LG5-10–15 and LG15-10–5 predominantly fall within the range of 0.8–1.2, where the values for LG5-10–15 are closer to 1. This means that LG5-10–15 has better impedance matching than LG15-10–5, allowing more EMWs to enter into the composite. On the other hand, the attenuation constant (*α*) is also important because it reflects the capacity of materials to attenuate the incoming EMWs, which can be calculated by formula S12. As shown in Fig. 3j, *α* is mainly depended on the absorbent content in composites with the exception of L15 due to its low *ε″* and *μ″*. By comparison, layered gradient composites reveal the higher *α* values than those of layered composites due to the presence of multilevel interfaces causing multiple reflections and scattering for EMWs. In particular, composite LG5-10–15 shows a higher *α* than LG15-10–5 at 8.2–11.5 GHz, indicating that the ascending gradient structure can cause more interface reflections between absorbents relative to the descending gradient structure, thus resulting in more EMW loss. Therefore, in view of the above electromagnetic parameter analysis, the layered gradient composites, especially ascending gradient composite (LG5-10–15), are expected to have better EMW absorbing properties than others.

### Electromagnetic Absorption Performance of Layered and Gradient Composites

**Fig. 4:**
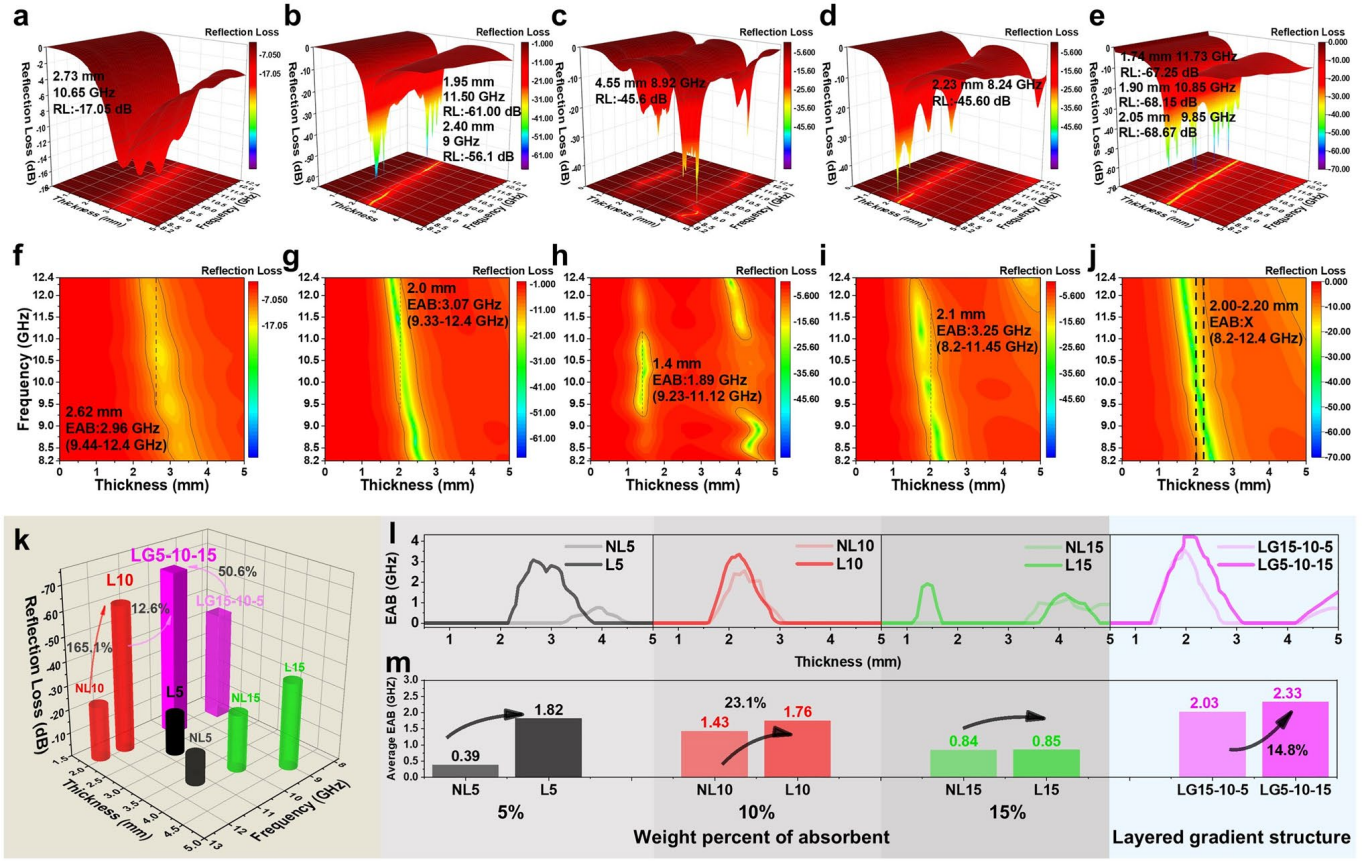
3D representations and 2D representations of *RL–f* of (**a, f**) L5, (**b, g**) L10, (**c, h**) L15, (**d, i**) LG15-10–5, and (**e, j**) LG5-10–15; **k** statistics *RL*_min_, **l** EAB values for various thickness and **m** average EAB for NL5, NL10, NL15, L5, L10, L15, LG15-10–5, and LG5-10–15

The EMW absorbing performance is usually expressed by the *RL* according to transmission line theory, which can be calculated from the correlation of ***ε***_*r*_ and ***μ***_*r*_, applying formulas S11 and S13. According to the 3D representations of *RL–f* of L5, L10, and L15 in Fig. 4a-c, the reflection loss capacity of composites initially increases and then decreases with the increase in the absorbent content; L10 exhibits the best reflection loss with *RL*_min_ of −61.00 dB at 11.5 GHz in 1.95 mm. It is noteworthy that the layered composites have better reflection loss capacity compared to non-layered composites (Fig. S12a-c). This is because the layered stacking extends the areas of interaction between 2D Ni@MXene and incident EMWs, causing more EMWs to be consumed. For the layered and gradient composites, LG15-10–5 exhibits a *RL*_min_ of -45.60 dB at 8.24 GHz in 2.23 mm (Fig. 4d), but its values at whole X-band are all lower than L10. By comparison, LG5-10–15 reveals best reflection loss with *RL*_min_ of −68.67 dB at 9.85 GHz in 2.05 mm (Fig. 4e). More intriguingly, LG5-10–15 composite exhibits multiple peaks *RL* within the thickness range of 1.7 to 2.5 mm, e.g., -67.25 dB at 11.73 GHz in 1.74 mm, -68.15 dB at 10.85 GHz in 1.90 mm, and -68.67 dB at 9.85 GHz in 2.05 mm.

Besides, the EAB representing the frequency range of 90% wave absorption (*RL* ≤ -10 dB) is equally important for EMW absorption performance. The non-layered composites exhibit the extremely narrow EAB with the best one for NL10 of EAB = 2.54 GHz (9.04–11.58 GHz) at a thickness of 2.30 mm (Fig. S12d-f). In contrast, the EABs of the layered composites are effectively improved with optimal one for L10 of EAB = 3.07 GHz (9.33–12.4 GHz) at a thickness of 2.00 mm (Fig. 4f-h). Importantly, the LG5-10–15 composite with the ascending layered gradient structure can effectively absorb and loss EMW at a wide frequency range with the wide EAB for entire X-band in thickness of 2.00–2.20 mm. The EAB of LG15-10–5 composite cannot completely cover the entire X-band, because the incident side L15 layer cannot form a good impedance match with air, causing most of the EMWs to be reflected, thereby reducing the EAB.

To visually demonstrate the advantages of layered gradient composite in EMW absorbing properties, *RL* and EAB are counted in Fig. 4k-m. In view of the best in each series composites, it can be found that the *RL*_min_ of L10 is increased by 165.1% as distribution of Ni@MXene in composites from non-layered to layered, with the further increase by 12.6% when Ni@MXene concentration presents the gradient distribution in layered composite (Fig. 4k). Moreover, the *RL*_min_ of the ascending gradient composite (LG5-10–15) increased by 50.6% compared to that of descending gradient composite (LG15-10–5). For the statistical variation of EAB with thickness, it can be seen that the layered distribution of Ni@MXene in composites significantly improves EAB compared with the non-layered composites (Fig. 4l), and the layered gradient composites have wider EAB than the layered composites. Compared to LG15-10–5, LG5-10–15 exhibits a broader area in EAB with a larger thickness range of 1.35–3.05 mm. The average EAB more clearly demonstrates the variation in EAB in the presence of the major peak EAB, which shows the significant increment from non-layered composites to layered composites (e.g., 23.1% from NL10 to L10), and from layered composites to layered gradient composites (32.4% from L10 to LG5-10–15). Moreover, the average EAB of LG5-10–15 increases by 14.8% when compared to LG15-10–5. Therefore, in terms of the great increments in *RL*_min_ and EAB for LG5-10–15, it can be concluded that EMW absorption properties can be greatly improved by layering arrangement of absorbents with ascending concentration gradient in composites. Compared with other works using the Ni@MXene as the absorbent through aspects such as absorbent content, *RL*_min_, EAB, and fitting thickness (Fig. S13 and Table S1), this work can achieve good EAB and considerable *RL*_min_ at relatively low absorbent content.

**Fig. 5:**
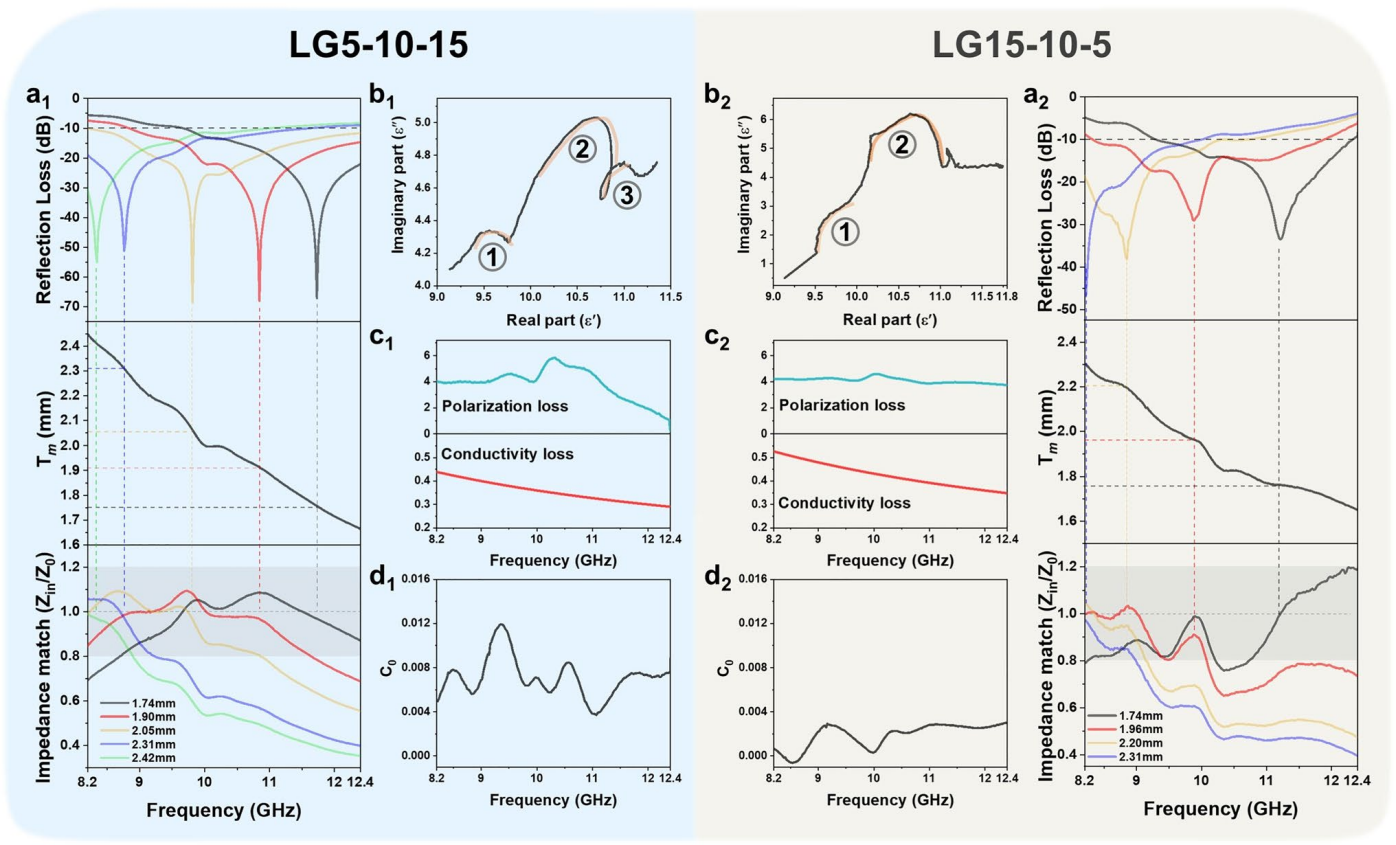
**a**_**1**_ *RL–f* curves, dependence of 1/4λ matching thickness on *RL* peak frequency, and corresponding impedance matching, **b**_**1**_ Cole–Cole curve and corresponding **c**_**1**_ polarization loss and conductivity loss, **d**_**1**_ C_0_–*f* curve of LG5-10–15. **a**_**2**_ *RL–f* curves, dependence of 1/4λ matching thickness on *RL* peak frequency, and corresponding impedance matching, **b**_**2**_ Cole–Cole curve and corresponding **c**_**2**_ polarization loss and conductivity loss, **d**_**2**_ C_0_–*f* curve of LG15-10–5

It is noteworthy that the absorption performance of composites is closely related to their thickness, where the *RL*_min_ and EAB are progressively shifted to lower frequencies with the increase in the thickness (Figs. 5a1, a2 and S14). This trend is consistent with the quarter wavelength (1/4*λ*) theory according to formula S14, i.e., when the thickness of the absorbing material reaches the theoretical thickness (*T*_m_) of quarter wavelength (1/4*λ*), the phase difference between incident and reflected waves is 180°, resulting in mutual interference and cancellation phenomenon. More importantly, the absorption peak corresponds to the position where impedance matching is closest to 1 (Figs. 5a1, a2 and S15), which achieves optimal impedance matching effect to allow more EMWs to enter [59, 60], thereby exerting the microwave loss capacity of the absorbent and achieving the maximum reflection loss. In contrast, based on the order of L5 as incident layer, L10 as loss layer, and L15 as reflection layer according to their permittivity, LG5-10–15 reveals the better impedance matching than LG15-10–5, thus showing the more optimal microwave absorption performance.

The loss mechanisms are also analyzed according to electromagnetic parameters. According to Debye relaxation theory [61–63] (formulas S15-S18), the Cole–Cole plots (Figs. 5b1, b2 and S16) reveal a greater number of semicircular rings for LG5-10–15 compared to LG15-10–5, indicating that the layered ascending gradient structure has more numerous polarization losses. Due to the fact that the EMWs incident from the surface of the composite LG15-10–5 with high absorbent content of 15%, the polarization in strong dielectric imaginary can keep up with the changes of the EMWs, thereby reducing a relaxation phenomenon. Generally speaking, the dielectric loss is closely related to polarization loss and conductivity loss. In order to quantitatively describe their contribution to dielectric loss, the formula S18 can be used to fit and obtain the polarization loss and conductivity loss (Figs. 5c1, c2 and S17) [64]. It can be observed that polarization loss is dominant compared to conductivity loss for all composites. The polarization loss can be highly enhanced by changing layered structure to layered and gradient structure as a result of the extra multilevel interfacial reflections and scattering of EMWs. Moreover, LG5-10–15 exhibits a higher polarization loss than LG15-10–5 due to the relatively weak reflection ability at inverse impedance mismatch interface. Besides, magnetic loss can occur between Ni@MXene and alternating electromagnetic fields, which is expressed in formulas S19-S23 according to ferromagnetic resonance theory. In layered composites, the magnetic loss strength is mainly dependent on the magnetic Ni@MXene content (Fig. S18). However, LG5-10–15 reveals the higher C_0_ value at whole frequency than LG15-10–5 (Fig. 5d1, d2), and the presence of more resonance peaks in LG5-10–15 indicates that more natural and exchange resonances will be generated when EMWs are incident into the layered gradient composite. Therefore, in terms of electromagnetic parameter analysis, LG5-10–15 not only has more impedance matching characteristics, but also shows higher dielectric and magnetic loss capacity compared with LG15-10–5.

### Simulated EMW Transmission and Microwave Absorption Mechanism of Layered and Gradient Composites

**Fig. 6:**
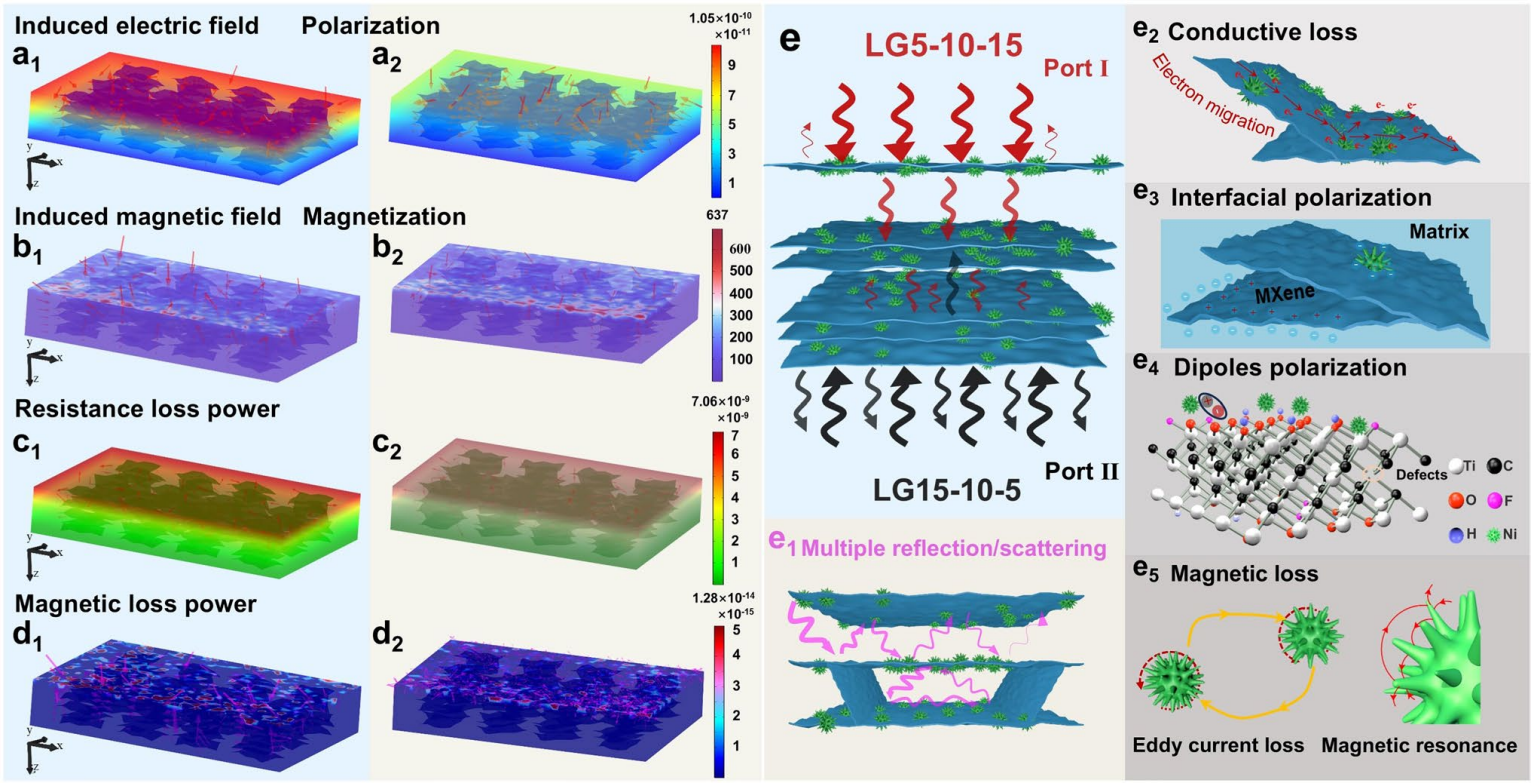
Distributions of electric field polarization (V m^−1^), magnetic field magnetization (A m^−1^), resistance loss power (W m^−3^), and magnetic loss power (W m^−3^) of (a1-d1) LG5-10–15 and (a2-d2) LG15-10–5. **e** Schematic illustration of microwave absorbing mechanism for layered and gradient Ni@MXene/PH composite at port I and port II

To further confirm the significant influence of the layered gradient distribution of absorbents on EMW absorption performance, the distribution of electric/magnetic field modes and energy loss in 10 GHz were simulated by using finite element analysis [65]. According to the different incidence directions of EMWs, the ideal simulation models of LG5-10–15 and LG15-10–5 are established in Fig. S20a, and the simulation method is outlined in Section S8 (Supporting Information). From Figs. 6a1, a2 and S20b, the intensities of both incident and induced electric fields gradually decrease from the incident region to the bottom, where LG5-10–15 exhibits superior polarization capability compared to LG15-10–5, resulting in a larger electric field difference and a stronger induced electric field. However, there is no significant difference in their magnetization ability as evidenced by the similar incident and induced magnetic fields (Figs. 6b1, b2 and S20c). The effect of the layered gradient distribution of the absorbent on the loss capacity of EMWs was analyzed by comparing the resistance/magnetic loss powers (Fig. 6c, d). The LG5-10–15 exhibits high resistance loss ability due to its good polarization ability in the induced electric field. On the contrary, LG15-10–5 with high impedance mismatch with air allows fewer EMWs to penetrate, leading to weaker resistance loss capability. Similarly, the magnetic loss power of LG5-10–15 is also greater than that of LG15-10–5.

Overall, the layered and gradient distribution of absorbents significantly influences the EMW absorbing performance of composites. In the case of LG5-10–15 (Fig. 6e), the surface layer with low absorbent concentration can achieve effective impedance match with air, enabling more EMWs to enter the interior of the composite from port I. The medium-concentration loss layer exerts the electromagnetic loss ability of absorbent. The reflector can further reflect EMWs due to its high absorbent content forming local conductive networks, and the reflected EMWs are subsequently reabsorbed by the absorbents in the intermediate absorption layer. However, the high concentration of absorbent cannot form an excellent impedance match with air, so that less EMWs enter the composite from port II; thus, the electromagnetic loss capacity of the absorbing layer cannot be exerted for LG15-10–5 composite. On the other hand, the layered arrangement of Ni@MXene in composite further enhances the electromagnetic loss ability of absorbent by creating numerous interaction areas with vertically incident EMWs to produce more polarization loss, inducing the multiple reflection/scattering to increase the propagation path of EMWs in composite, and further enhances the attenuation effect of absorbents. Meanwhile, the highly oriented Ni@MXene creates local conductive pathways that facilitate electron migration and enhance the conductivity loss capability. Numerous heterogeneous interfaces are formed between MXene, Ni particles, and the PVDF-HFP matrix, leading to strong interface polarization losses when interacting with EMWs. The numerous functional groups on the surface and internal defects of Ni@MXene and the polar fluorine atoms in matrix can lead to dipole polarization loss when EMWs arrive. Moreover, eddy current losses and magnetic resonance (natural resonance and exchange resonance) occur due to the presence and anisotropic needling of magnetic particles in the alternating electromagnetic field [66]. Therefore, combining the microscopic electromagnetic losses of absorbent and the enhanced loss effect caused by the macroscopic structure distribution, the layered and gradient LG5-10–15 composite shows an excellent EMW absorbing performance.

## Conclusion

In summary, the layered gradient structure of magnetic MXene integral absorbing material was prepared through the microstructure design, layered structure arrangement of absorbents, and gradient concentration distribution of composite. Initially, the introduction of magnetic Ni particles onto the surface of MXene endows the capability of electromagnetic synergistic loss and regulates effectively the electromagnetic parameters of absorbents. Secondly, the layered arrangement of Ni@MXene in composite can increase the interaction area between absorbents and EMWs, and induce the multiple reflection/scattering for incident EMWs, so that the L10 composite has superior reflection loss with *RL*_min_ of − 61.00 dB at 11.5 GHz in 1.95 mm. Furthermore, the gradient concentration distribution of absorbents effectively modulates the impedance matching of composites, allowing more EMWs to enter the composite and undergo multiple reflection/scattering, thereby improving their EAB. The results have demonstrated that LG5-10–15 not only exhibits superior reflective loss capabilities (− 68.67 dB at 9.85 GHz in 2.05 mm) but also achieves complete coverage of the X-band within the thickness range of 2.00–2.20 mm for EAB compared to LG15-10–5. The microstructure design of absorbents and the preparation of layered gradient at the macroscale have been proposed in this work to obtain integral absorbing materials for achieving strong and broadband microwave absorption.

## Supplementary Information

Below is the link to the electronic supplementary material.

Supplementary file1 (DOCX 14464 KB)
