# Supplementary material for: Absorption–Reflection–Transmission Power Coefficient Guiding Gradient Distribution of Magnetic MXene in Layered Composites for Electromagnetic Wave Absorption
Source: Nanomicro Lett. 2025 Feb 17;17:147. doi: 10.1007/s40820-025-01675-7 (PMC11832828; doi:10.1007/s40820-025-01675-7)
Supplement: Supplementary file 1 — Supplementary file1 (DOCX 14464 KB) [file 40820_2025_1675_MOESM1_ESM.docx]

Supporting Information for

**Absorption-Reflection-Transmission Power Coefficient Guiding Gradient Distribution of Magnetic MXene in Layered Composites for Electromagnetic Wave Absorption**

Yang Zhou^1^, Wen Zhang^1^, Dong Pan^1^, Zhaoyang Li^1^, Bing Zhou^1^, Ming Huang^1*^, Liwei Mi^1^,^2^, Chuntai Liu^1^, Yuezhan Feng^1*^, Changyu Shen^1^

^1^State Key Laboratory of Structural Analysis, Optimization and CAE Software for Industrial Equipment, National Engineering Research Center for Advanced Polymer Processing Technology, Zhengzhou University, Zhengzhou 450002, P. R. China.

^2^Yaoshan Laboratory, Pingdingshan University, Pingdingshan 467000, P. R. China

^*^Corresponding authors. E-mail: [huangming@zzu.edu.cn](mailto:huangming@zzu.edu.cn) (Ming Huang), [yzfeng@zzu.edu.cn](mailto:yzfeng@zzu.edu.cn) (Yuezhan Feng)

**S1 Filler Orientation Test**

To assess the orientation of nanosheets with parallel alignment, Wide-angle X-ray scattering was utilized. The formulas are as follows [S1, S2]:

$f=\frac{3\langle\cos^{2}\theta\rangle-1}{2}$ (S1)

$\langle\cos^{2}\theta\rangle=\frac{\int_{0}^{\pi/2} I(\theta)\cos^{2}\theta\sin\theta d\theta}{\int_{0}^{\pi/2} I(\theta)\sin\theta d\theta}$ (S2)

where $\langle\cos^{2}\theta\rangle$ represents the average value of the squared azimuthal cosine for the (002) peak of the tested MXene flakes, and $I(\theta)$ denotes the signal intensity at an azimuthal angle of $\theta$.

**S2** **A-R-T Coefficients and EMI SE**

The four parameters (**S**_11_, **S**_21_, **S**_22_, **S**_12_) containing eight data were obtained using the vector network analyser. Among them, the A_21_, R_21_, T_21_ coefficients of port Ⅰ are calculated as follows S3-S4; The calculation formula for their SE_R_21, SE_A_21, SE_T_21 shown in S5-S6 [S3-S5]. Similarly, the A_12_, R_12_, T_12_, SE_R_12, SE_A_12, and SE_T_12 of port Ⅱ can be obtained according to formula S7-S10.

$R_{21}=\left|S_{11}\right|^{2}$, $T_{21}=\left|S_{21}\right|^{2}$ (S3)

$A_{21}=1-R_{21}-T_{21}$ (S4)

$SE_{R}21=-10\lg\left(1-R_{21}\right)$, $SE_{A}21=-10\lg\left(\frac{T_{21}}{1-R_{21}}\right)$ (S5)

$SE_{T}21=SE_{R}21+SE_{A}21+SE_{M}21$ (S6)

$R_{12}=\left|S_{22}\right|^{2}$, $T_{12}=\left|S_{12}\right|^{2}$ (S7)

$A_{12}=1-R_{12}-T_{12}$ (S8)

$SE_{R}12=-10\lg\left(1-R_{12}\right)$, $SE_{A}12=-10\lg\left(\frac{T_{12}}{1-R_{12}}\right)$ (S9)

$SE_{T}12=SE_{R}12+SE_{A}12+SE_{M}12$ (S10)

**S3 Impedance Matching and Attenuation Constant**

Impedance matching characteristics are represented by |**Z**_in_/**Z**_0_|. The attenuation constant (α) is used to evaluate the attenuation ability of the composites. Optimal impedance matching is achieved when the *|***Z**_in_/**Z**_0_*|* value approaches or Eq. 1 [S6]. The higher attenuation constant indicates greater absorption of electromagnetic waves by the material. |**Z**_in_/**Z**_0_| and α can be calculated as follows [S7]:

$Z_{in}=Z_{0}\sqrt{\frac{\mu_{r}}{\varepsilon_{r}}}tanh\left(j\frac{2\pi fd\sqrt{\mu_{r}\varepsilon_{r}}}{c}\right)$ (S11)

$\alpha=\frac{\sqrt{2}\pi f}{c}\times\sqrt{\left(\mu''\varepsilon''-\mu'\varepsilon'\right)+\sqrt{\left(\mu''\varepsilon''-\mu'\varepsilon'\right)^{2}+\left(\mu'\varepsilon''+\mu''\varepsilon'\right)^{2}}}$ (S12)

Where ${\text{ }\text{Z}}_{\text{in}}$ is the input impedance, $\text{Z}_{0}$ is the impedance of free space, $\text{ε}_{\text{r}}$ is the relative complex permittivity ($\text{ε}_{\text{r}}=\varepsilon^{'}-j\varepsilon^{''}$), $\text{μ}_{\text{r}}$ is the relative complex permeability ($\text{μ}_{\text{r}}=\mu^{'}-j\mu^{''}$), *d*, *f*, and c are thickness, frequency, and the velocity of EMWs in free space, respectively.

**S4 Electromagnetic Wave Absorption Measurements**

The vector network analyser was employed to measure the relative complex permittivity and relative permeability across the frequency range of 8.2-12.4 GHz. Further, the measured EM parameters were used to calculate the *RL-f* curves according to the following formula [S8, S9]:

$RL=20\log_{10}\left|\left(Z_{in}-Z_{0}\right)/\left(Z_{in}+Z_{0}\right)\right|$ (S13)

The higher the value of the *RL*, the stronger EMWs absorption capacity.

**S5 1/4λ Matching Thickness Interference Loss**

According to the homogeneous plate model, when the thickness of the material is an odd multiple of a quarter of the wavelength, the phase difference between the incident wave and the reflected wave of EMWs by the baffle at the interface of the absorbent is about 180^o^, EMWs are then dissipated by interference cancellation, which is the fourth-wavelength cancellation theory [S10, S11]:

$t_{m}=\frac{n\lambda}{4}=\frac{nc}{4f_{m}\sqrt{\left|\varepsilon_{r}\right|\left|\mu_{r}\right|}}$ **（**$n$=1,3,5,...**）** (S14)

Therefore, for any specific frequency of EMWs, the corresponding material thickness can be designed according to the quarter-wavelength theory.

**S6 Debye Relaxation**

The polarization phenomenon of materials involves the movement of bound charges along the direction of the applied electric field, resulting in electron displacement or dipole orientation. In high-frequency electromagnetic fields, electron motion or dipole deflection may not fully follow the electric field changes, resulting in a hysteresis effect in electron or dipole polarization relaxation [S12]. Based on the Debye relaxation theory, the Cole-Cole semicircular equation can well represent the relaxation process in electromagnetic fields [S13, S14]:

$\varepsilon'=\varepsilon_{\infty}+\frac{\varepsilon_{s}-\varepsilon_{\infty}}{1+\omega^{2}\tau^{2}}$ (S15)

$\varepsilon''=\frac{\varepsilon_{s}-\varepsilon_{\infty}}{1+\omega^{2}\tau^{2}}\omega\tau+\frac{\sigma}{\omega\varepsilon_{0}}$ (S16)

$\varepsilon_{\infty}$, $\varepsilon_{s}$, $\omega$, $\tau$, $\sigma$ and $\varepsilon_{0}$ are the relative permittivity at high frequency limit, the static permittivity, the angular frequency, the polarization relaxation time, the conductivity, and the vacuum permittivity. From Eqs. S15and S16, the relationship between ε' and ε'' can be derived as follow:

$\left(\varepsilon'-\frac{\varepsilon_{s}+\varepsilon_{\infty}}{2}\right)^{2}+\left(\varepsilon''\right)^{2}=\left(\frac{\varepsilon_{s}-\varepsilon_{\infty}}{2}\right)^{2}$ (S17)

In the plots, each Cole-Cole semicircle corresponds to a Debye relaxation process. When the locus of the permittivity in the complex plane is a semicircle with endpoints on the axis of reals and centre below this axis a polarization behavior is formed. For polarizations that can keep up with changes in electromagnetic fields, there will be no semi-circular ring (relaxation phenomenon). The straight line at the end of the curves indicates that dielectric loss predominantly arises from conduction losses.

$\varepsilon''=\varepsilon''_{\text{p}}+\varepsilon''_{c}=(\varepsilon_{S}-\varepsilon_{\infty})\frac{2\pi f\tau}{1+(2\pi f)^{2}\tau^{2}}+\frac{\sigma}{2\pi f\varepsilon_{0}}$ (S18)

$\varepsilon^{''}$ can be obtained through a vector network analyzer, and it is also related to conductivity (σ), so polarization loss and conductivity loss can be obtained through nonlinear fitting using formula S18 [S15].

**S7 Magnetic Loss Factors**

Magnetic loss results from the interaction between magnetic absorbents and electromagnetic fields. Magnetic loss primarily results from natural resonance, exchange resonance, eddy current losses, magnetic coupling effects, and so on [S16]. According to the ferromagnetic resonance theory, the natural resonance frequency can be expressed as follows [S17]:

$2\pi f_{r}=\gamma H_{a}$ (S19)

$H_{a}=4\left|K_{1}\right|/3\mu_{0}M_{s}$ (S20)

$K_{1}=\mu_{0}M_{s}H_{c}/2$ (S21)

where $f_{r}$, $\gamma$, $H_{a}$, $K_{1}$, $\mu_{0}$, $M_{s}$ and $H_{c}$ the natural resonance frequency, the gyromagnetic ratio, anisotropy energy, the anisotropy coefficient, the vacuum permeability, saturation magnetization and is coercive force. Natural resonance and exchange resonance are the general forms of high frequency magnetic resonance. Natural resonance usually occurs at 2-10 GHz, whereas exchange resonance usually occurs at higher frequency (>10 GHz).

Eddy current loss is the joule heat or energy loss caused by eddy current like water swirling around a good conductor to hinder the transmission of electromagnetic waves under the action of an alternating magnetic field. In general, the relationship between the eddy current loss, conductivity ($\sigma$) and the matching thickness ($d$) of a magnetic material can be expressed as [S18]:

$\mu''=2\pi\mu_{0}\left(\mu'\right)d^{2}f\sigma$ (S22)

where $\mu_{0}$ is the vacuum permeability, and the eddy current induction formula can be expressed as [S15]:

$C_{0}=\mu''\left(\mu'\right)^{-2}f^{-1}=2\pi\mu_{0}\sigma d^{2}/3$ (S23)

If the magnetic loss is caused only by the eddy current loss mechanism, the value of the $C_{0}$-$f$ curve is constant.

**S8 Electromagnetic Simulation Method**

COMSOL Multiphysics software was used to simulate RL for the layered gradient magnetic MXene composites. The radio frequency mode was selected use finite element simulation programs. In the perfectly matched layer of Fig. S20a, an electromagnetic wave with a frequency of 10 GHz was imported and corresponding permittivity and permeability data were selected in Fig. 3e-h. The wave transmission is shown as follows:

$\nabla\times\mu_{r}^{-1}\left(\nabla\times E\right)-k_{0}^{2}\left(\varepsilon_{r}-\frac{j\sigma}{\omega\varepsilon_{0}}\right)E=0$ (S24)

$E\left(x,y,z\right)=\widetilde{E}\left(x,y\right)e^{-ik_{Z}Z}$ (S25)

Floquet periodic boundary conditions:

$E_{dst}=E_{src}e^{-ik_{F}\cdot\left(r_{dst}-r_{src}\right)}$ (S26)

$H_{dst}=H_{src}e^{-ik_{F}\cdot\left(r_{dst}-r_{src}\right)}$ (S27)

The port of the electric field:

$S=\frac{\int_{\partial\Omega} \left(E-E_{1}\right)\cdot E_{1}}{\int_{\partial\Omega} E_{1}\cdot E_{1}}$ (S28)

The port of the magnetic field:

$S=\frac{\int_{\partial\Omega} \left(H-H_{1}\right)\cdot H_{1}}{\int_{\partial\Omega} H_{1}\cdot H_{1}}$ (S29)

the scattering boundary condition:

$n\times\left(\nabla\times\left(E\right)\right)\text{-}jkn\times\left(E\times n\right)\text{=}0$ (S30)

Ideal electrical conductor:

$n\times E\text{=}0$ (S31)

The typical wavelength definition of a perfectly matched layer in a fictional domain is:

$2*pi/abs(emw.k_{0}*cos(theta))$ (S32)

Among, **E**, **H**, *k*_0_, $\boldsymbol{\varepsilon}_{r}$, $\sigma$, $\omega$, $\varepsilon_{0}$, and theta represent the electric field intensity (V m^-1^), the magnetic field intensity (A m^-1^), the free space wave number (rad m^-1^), the complex dielectric constant (F m^-1^), the conductivity (S m^-1^), the vacuum dielectric constant (8.8542×10^-12^ F m^-1^) and incidence angle (rad), respectively.

The absorbent model (Fig. S19a) was established for MXene with a length of 10 μm, a width of 8 μm, and a thickness of 2 nm. The spherical radius of Ni particles is 200 nm. Figure S19b is the cross-sectional view of the increasing layer structure of the absorbent in a single absorbing unit. The model (Fig. S20a) was established with grey part, green balls, transparent parts, bright yellow part representing the cross-section of MXene 2D nanosheets, the nickel particles attached to the 2D nanosheets, the polymer matrix and the air layer, respectively. The upper edge of the air layer is a perfectly matched layer (PML) and an electric or magnetic field source port, the bottom edge is the scattering boundary layer (SBL) condition, and both sides are Floquet periodic boundary conditions. The EMW excitation source is the electric field polarization source or the magnetic field magnetization source along the Z-direction with an incident power set to 1 W m^-1^. The electric field and magnetic field at the excitation source are vector orthogonal, and their phase difference is π/2. The models were established as LG5-10-15 and LG15-10-5 from layered ascending concentration gradient composite and layered descending concentration gradient composite based on magnetic MXene fillers. The ideal simulation models are shown in Fig. S20a. The electric and magnetic field intensity distributions are represented by different colors in Fig. S20b-c, where the black arrow represents the field density vector/magnetic flux density.

**S9 Supplementary Figures**

**Fig. S1** SEM image of (**a**) MXene, (**b**) Ni and (**c**) Ni@MXene

**Fig. S2** XPS spectra of (**a**) MXene and Ni@MXene, MXene and Ni@MXene at the (**b**) Ti 2p (**c**) O 1s and (**d**) C 1s region

**Fig. S3** FT-IR spectra of Ni, MXene and Ni@Mxene

**Fig. S4** Digital photographs of (**a**) Ni, (**b**) MXene, and (**c**) Ni@MXene adsorbed by the magnet

**Fig. S5** 2D-WAXD images of (**a**) NL5, (**b**) NL10, (**c**) NL15, (**d**) L5, (e) L10

**Fig. S6** (**a**) Breaking strength and elongation at break, (**b**) toughness of NL5, NL10, NL15, L5, L10, L15 composites

**Fig. S7** Magnetic hysteresis loops of L5, L10, L15, LG at room temperature

**Fig. S8** SE_T_, SE_A_, SE_R_ of (**a**) L5, (**b**) L10, (**c**) L15, (**d**) LG15-10-5 (port Ⅱ) and LG5-10-15 (port Ⅰ) composites and their corresponding (e-h) average statistical values

**Fig. S9** (**a-c**) SE_T_, SE_A_, SE_R_ and their corresponding (**d-f**) statistical charts, (**g-i**) A-R-T coefficients and their corresponding (**j-l**) statistical charts of NL5, NL10, NL15

**Fig. S10** (**a**) Tan*δ_ε_* and (**b**) Tan*δ_μ_* of L5, L10, L15, LG15-10-5 and LG5-10-15

**Fig. S11** (**a**) Real part permittivity, (**b**) imaginary part permittivity, (**c**) Tan*δ_ε_*, (**d**) real part permeability, (**e**) imaginary part permeability, (**f**) Tan*δ_μ_* of NL5, NL10, NL15

**Fig. S12** (**a-c**) Three-dimensional representations and (**d-f**) two-dimensional representations of *RL*-*f* of NL5, NL10, NL15

**Fig. S13** Comparison of basic parameters of other absorbing materials according to Table S1

**Fig. S14** (**a-c**) RL-*f* curves of L5, L10, L15

**Fig. S15** Impedance matching curves of (**a**) L5, (**b**) L10, (**c**) L15

**Fig. S16** Cole-Cole curves of (**a**) L5, (**b**) L10, (**c**) L15

**Fig. S17** (**a**) Polarization loss and (**b**) conductivity loss of L5, L10, L15

**Fig. S18** C_0_-*f* curves of L5, L10, L15

**Fig. S19** (**a**) The size of a single absorbent, (**b**) the layer structure of a single unit

**Fig. S20** (**a**) Idealized simulation model, (**b**) electric field mode (V m^-1^), (**c**) magnetic field mode (A m^-1^) of LG5-10-15 and LG15-10-5

**Table S1** Comparison of basic parameters of other absorbing materials

|  | Absorbing materials | content/% | RL_min_/dB | EAB  /GHz | Thickness  /mm | References |
| --- | --- | --- | --- | --- | --- | --- |
| S1 | Magnetic MXene | 10 | -52.6 | 3.7 | 3 | [S19] |
| S2 | MXene@Ni | 79.5 | -59.6 | 4.48 | 1.5 | [S20] |
| S3 | MXene-CNTs/Ni | 30 | -56.4 | 3.95 | 2.4 | [S21] |
| S4 | Ti_3_C_2_T_x_/Ni | 50 | -47.06 | 3.6 | 1.5 | [S22] |
| S5 | Ti_3_C_2_T_x_ and NiCo_2_O_4_ | 50 | -50.96 | 2 | 2.18 | [S23] |
| S6 | NiFe_2_O_4_/Ti_3_C_2_T_x_ | 3 | -41.83 | 3.52 | 3 | [S24] |
| S7 | MXene/Ni/C | 57 | -42.3 | 5.6 | 5 | [S25] |
| S8 | Ti_3_C_2_T_x_/nickel | 40 | -47.34 | 4.65 | 1.5 | [S26] |
| S9 | MXene/Ni_0.6_Zn_0.4_Fe_2_O_4_ | 30 | -66.2 | 4.74 | 1.629 | [S27] |
| S10 | MXene/MnO_2_/Ni | 30 | -54.4 | 6.08 | 2.67 | [S28] |
| S11 | Ti_3_C_2_/Ni | 60 | -24.3 | 3.6 | 2.2 | [S29] |
| S12 | Ni@MXene | 10 | -68.67 | 4.2 | 2.05 | This work |

**Supplementary References**

1. J. Yu, H. Cheng, Y. Wang, C. He, B. Zhou et al., Multiple shearing-induced high alignment in polyethylene/graphene films for enhancing thermal conductivity and solar-thermal conversion performance. Chem. Eng. J. **480**, 148062 (2024). <https://doi.org/10.1016/j.cej.2023.148062>
2. C. Chen, G. Quek, H. Liu, L. Bannenberg, R. Li et al., High-rate polymeric redox in MXene-based superlattice-like heterostructure for ammonium ion storage. Adv. Energy Mater. **14**, 2402715 (2024). <https://doi.org/10.1002/aenm.202402715>
3. A.A. Isari, A. Ghaffarkhah, S.A. Hashemi, S. Wuttke, M. Arjmand, Structural design for EMI shielding: from underlying mechanisms to common pitfalls. Adv. Mater. **36**, 2310683 (2024). <https://doi.org/10.1002/adma.202310683>
4. X. Liu, H. Liu, H. Wu, Q. Zhou, H. Liang et al., Structural electromagnetic absorber based on MoS_2_/PyC-Al_2_O_3_ ceramic metamaterials. Small **19**, 2300664 (2023). <https://doi.org/10.1002/smll.202300664>
5. T. Yun, H. Kim, A. Iqbal, Y.S. Cho, G.S. Lee et al., Electromagnetic shielding of monolayer MXene assemblies. Adv. Mater. **32**, 1906769 (2020). <https://doi.org/10.1002/adma.201906769>
6. W. Luo, M. Wang, K. Wang, P. Yan, J. Huang et al., A robust hierarchical MXene/Ni/aluminosilicate glass composite for high-performance microwave absorption. Adv. Sci. **9**, 2104163 (2022). <https://doi.org/10.1002/advs.202104163>
7. M. Ling, F. Ge, F. Wu, L. Zhang, Q. Zhang et al., Effect of crystal transformation on the intrinsic defects and the microwave absorption performance of Mo_2_TiC_2_Tx/RGO microspheres. Small **20**, 2306233 (2024). <https://doi.org/10.1002/smll.202306233>
8. Y. Zhang, L. Pan, P. Zhang, Z. Sun, Gradient multilayer design of Ti_3_C_2_T_x_ MXene nanocomposite for strong and broadband microwave absorption. Small Sci. **2**, 2200018 (2022). <https://doi.org/10.1002/smsc.202200018>
9. X. Su, J. Wang, T. Liu, Y. Zhang, Y. Liu et al., Controllable atomic migration in microstructures and defects for electromagnetic wave absorption enhancement. Adv. Funct. Mater. **34**, 2403397 (2024). <https://doi.org/10.1002/adfm.202403397>
10. Y. Cheng, X. Sun, Y. Yuan, S. Yang, Y. Ning et al., Flexible SiO_2_/rGO aerogel for wide-angle broadband microwave absorption. Carbon **217**, 118580 (2024). <https://doi.org/10.1016/j.carbon.2023.118580>
11. G. Qin, X. Huang, X. Yan, Y. He, Y. Liu et al., Carbonized wood with ordered channels decorated by NiCo_2_O_4_ for lightweight and high-performance microwave absorber. J. Adv. Ceram. **11**, 105–119 (2022). <https://doi.org/10.1007/s40145-021-0520-z>
12. A. Elhassan, J. Li, I. Abdalla, Z. Xu, J. Yu et al., Ant-nest-inspired biomimetic composite for self-cleaning, heat-insulating, and highly efficient electromagnetic wave absorption. Adv. Funct. Mater., 2407458 (2024). <https://doi.org/10.1002/adfm.202407458>
13. H. Cheng, Y. Pan, W. Li, C. Liu, C. Shen et al., Facile design of multifunctional melamine foam with Ni-anchored reduced graphene oxide/MXene as highly efficient microwave absorber. Nano Today **52**, 101958 (2023). <https://doi.org/10.1016/j.nantod.2023.101958>
14. Y. Lou, J. Li, X. Li, Z. Zhu, Z. Shi et al., Single-atom Zn confined in hierarchical hollow microstructure as an acid/base-resistant microwave absorption materials. Nano Res. **17**, 6785–6794 (2024). <https://doi.org/10.1007/s12274-024-6689-z>
15. Z. Yao, S. Xu, X. Zhang, J. Yuan, C. Rong et al., CuCo nanocube/N-doped carbon nanotube composites for microwave absorption. ACS Appl. Nano Mater. **6**, 1325–1338 (2023). <https://doi.org/10.1021/acsanm.2c04972>
16. F. Hu, H. Tang, F. Wu, P. Ding, P. Zhang et al., Sn whiskers from Ti_2_SnC max phase: bridging dual-functionality in electromagnetic attenuation. Small Methods **8**, e2301476 (2024). <https://doi.org/10.1002/smtd.202301476>
17. L. Yao, Y. Wang, J. Zhao, Y. Zhu, M. Cao, Multifunctional nanocrystalline-assembled porous hierarchical material and device for integrating microwave absorption, electromagnetic interference shielding, and energy storage. Small **19**, e2208101 (2023). <https://doi.org/10.1002/smll.202208101>
18. S. Tian, Z. Sun, H. Ding, Z. Guo, P. Wang et al., Coordination environment-mediated different heteroatomic configuration from doping strategy for enhancing microwave absorption. Adv. Funct. Mater. **34**, 2310475 (2024). <https://doi.org/10.1002/adfm.202310475>
19. L. Liang, R. Yang, G. Han, Y. Feng, B. Zhao et al., Enhanced electromagnetic wave-absorbing performance of magnetic nanoparticles-anchored 2D Ti_3_C_2_T *_x_* MXene. ACS Appl. Mater. Interfaces **12**, 2644–2654 (2020). <https://doi.org/10.1021/acsami.9b18504>
20. C. Wen, X. Li, R. Zhang, C. Xu, W. You et al., High-density anisotropy magnetism enhanced microwave absorption performance in Ti_3_C_2_T*_x_* MXene@Ni microspheres. ACS Nano **16**, 1150–1159 (2022). <https://doi.org/10.1021/acsnano.1c08957>
21. X. Li, W. You, C. Xu, L. Wang, L. Yang et al., 3D seed-germination-like MXene with *in situ* growing CNTs/Ni heterojunction for enhanced microwave absorption *via* polarization and magnetization. Nano-Micro Lett. **13**, 157 (2021). <https://doi.org/10.1007/s40820-021-00680-w>
22. N. Li, X. Xie, H. Lu, B. Fan, X. Wang et al., Novel two-dimensional Ti_3_C_2_T_X_/Ni-spheres hybrids with enhanced microwave absorption properties. Ceram. Int. **45**, 22880–22888 (2019). <https://doi.org/10.1016/j.ceramint.2019.07.331>
23. T. Hou, B. Wang, M. Ma, A. Feng, Z. Huang et al., Preparation of two-dimensional titanium carbide (Ti_3_C_2_T_x_) and NiCo_2_O_4_ composites to achieve excellent microwave absorption properties. Compos. Part B Eng. **180**, 107577 (2020). <https://doi.org/10.1016/j.compositesb.2019.107577>
24. Y. Guo, D. Wang, T. Bai, H. Liu, Y. Zheng et al., Electrostatic self-assembled NiFe_2_O_4_/Ti_3_C_2_T_x_ MXene nanocomposites for efficient electromagnetic wave absorption at ultralow loading level. Adv. Compos. Hybrid Mater. **4**, 602–613 (2021). <https://doi.org/10.1007/s42114-021-00279-0>
25. J. Song, Y. Shen, G. Zhang, M. Sun, M. Han et al., Tunable interlayer spacings Ti_3_C_2_T_x_ MXene/Ni/C for enhanced electromagnetic microwave absorption. Ceram. Int. **50**, 41426–41435 (2024). <https://doi.org/10.1016/j.ceramint.2024.07.459>
26. L.-L. Zha, X.-H. Zhang, J.-H. Wu, J.-J. Liu, J.-F. Lan et al., Enhanced electromagnetic wave absorption based on Ti_3_C_2_T_x_ loaded nickel nanoparticles *via* polydopamine connection. Ceram. Int. **49**, 20672–20681 (2023). <https://doi.org/10.1016/j.ceramint.2023.03.198>
27. S. Guo, H. Guan, Y. Li, Y. Bao, D. Lei et al., Dual-loss Ti3C2Tx MXene/Ni_0.6_Zn_0.4_Fe_2_O_4_ heterogeneous nanocomposites for highly efficient electromagnetic wave absorption. J. Alloys Compd. **887**, 161298 (2021). <https://doi.org/10.1016/j.jallcom.2021.161298>
28. X. Wu, J. Huang, H. Gu, N. Li, Y. Wang et al., Ternary MXene/MnO_2_/Ni composites for excellent electromagnetic absorption with tunable effective absorption bandwidth. J. Alloys Compd. **911**, 165122 (2022). <https://doi.org/10.1016/j.jallcom.2022.165122>
29. Y. Liu, S. Zhang, X. Su, J. Xu, Y. Li, Enhanced microwave absorption properties of Ti_3_C_2_ MXene powders decorated with Ni particles. J. Mater. Sci. **55**, 10339–10350 (2020). <https://doi.org/10.1007/s10853-020-04739-8>
